# Why Your Body Can Jog Your Mind

**DOI:** 10.3389/fpsyg.2017.00362

**Published:** 2017-03-09

**Authors:** Manuela Macedonia, Claudia Repetto

**Affiliations:** ^1^Information Engineering, Johannes Kepler Universität LinzLinz, Austria; ^2^Neural Mechanisms of Human Communication, Max-Planck Institute for Human Cognitive and Brain SciencesLeipzig, Germany; ^3^Psychology, Università Cattolica del Sacro CuoreMilan, Italy

**Keywords:** embodiment, gesture theory, physical exercise, learning and memory, educational method, body and mind

Philosophical tradition influences the way we think about body and mind (Rogers, 1936). We have a body to move around and a mind to think and to learn (Descartes, 1637). At school, we sit, listen, and read, but we are not allowed to move. However, cognitive science has shown that our body is tightly linked to the mind (Wilson, 2002; Pecher and Zwaan, 2005; Gärtner, 2013). In this paper, we provide evidence that better learning is achieved if the body supports the mind. We review studies showing how physical movement impacts brain functions and structures, and why physical movement is beneficial to learning. Thereafter, we explain how the body supports the mind in difficult cognitive tasks. Finally, we discuss how the body can be employed as a tool in second language learning and mathematics.

## Body and mind and the brain?

The dichotomy body and mind grounds on the influential contribution of Descartes. In his Meditations of First Philosophy (1637), the French philosopher doubts on the existence of the body. The fact that he himself can think is his proof of the existence of his mind: *cogito ergo sum* (I think, therefore I am). Along this line, philosophy degrades the body to *rex extensa* (literally extended thing). The body is matter with organs and blood but ontologically distinct from the mind, the sole player in thinking. Interestingly, philosophical theories of the twentieth century have rejected this distinction (Shilling, 2012). Nonetheless, the dichotomy between body and mind is still well entrenched not only in everyday thinking but unfortunately also in education.

In Western countries, this separation biases educational methods and how people learn. Most schools make pupils sit during lessons. While the mind is doing its job, the body is doomed to rest, to exercise subordinate functions, no more than providing the organic facilities for the mind to exist. However, it is undeniable that the body cannot only be a mere container for organs. Stroke or neurodegeneration alter or destroy the brain, hence the mind. Similarly, diseases of the mind are dysfunctions of the brain, for example in neurotransmitter release (Fakhoury, 2015).

## Physical activity shapes the brain

It is well known that physical activity has positive effects on the body. However, it is not taken for granted that physical activity also benefits brain functions. Walking, cycling, and coordination training have a positive impact on academic achievement (Rasberry et al., 2011; Hötting and Röder, 2013). This is due to a number of mechanisms, as we describe below.

First, physical activity induces **neurogenesis** (from ancient Greek, the “birth of neurons”). Neurogenesis is a self-regeneration mechanism of the brain. Cellular loss and enhanced demand of information processing trigger the production of brain cells. After birth, neurogenesis mainly occurs in the hippocampus (Lieberwirth et al., 2016). From there, neural stem cells migrate and differentiate in other areas of the brain (Genin et al., 2014) and are activated upon learning (Kee et al., 2007). New cells raise the gray matter density and consequently, if stimulated and therefore connected, they enhance cognitive performance (Frangou et al., 2004; Narr et al., 2007).

Second, physical activity triggers the release of Brain Derived Neurotropic Factor (BDNF), a neurotrophin (protein) that encourages a large number of growth processes in the brain, first of all neurogenesis. Also, BDNF mediates the growth of dendrites, spine density, and synapses (Knaepen et al., 2010; Mang et al., 2016). Studies have shown a connection between BDNF and learning performance. One study tested the effect of ergometric bicycling on memory for French vocabulary. The German natives had to learn a set of words while cycling and another set during sedentary learning. Better results were found in memory performance for words acquired during cycling. Furthermore, higher performance correlated with higher BDNF in serum (Schmidt-Kassow et al., 2013). In a similar study, compared to sitting and listening, treadmill walking improved long-term retention of French vocabulary (Schmidt-Kassow et al., 2014).

Third, physical activity has an influence on two brain structures that mediate short-term memory, i.e., the **hippocampus** (Killgore et al., 2013) and the entorhinal cortex (Whiteman et al., 2016). Exercise induces growth of their volumes and this correlates with enhanced memory performance (Erickson et al., 2011; Sayal, 2015). In the long term, hippocampus size is related to academic performance (Chaddock-Heyman et al., 2014).

Fourth, physical activity has a positive impact on **cognitive control** (Guiney and Machado, 2013). Cognitive control includes a set of executive functions essential to human behaviors such as attention, reasoning, planning, problem solving, decision making, cognitive flexibility, inhibitory control, and short-term memory (Diamond, 2013). Executive functions develop during childhood and adolescence, and decline with age. Brain regions involved in cognitive control include the orbitofrontal cortex, the dorsolateral prefrontal cortex, and the anterior cingulate cortex. Larger volume and greater thickness of prefrontal cortex areas are associated with better executive performance (Weinstein et al., 2012; Yuan and Raz, 2014). Exercise in childhood and adolescence induces positive changes in brain tissue in these areas and therefore exercise is beneficial to cognitive functions, as reported in a meta-analysis by Verburgh et al. (2013).

Fifth, physical exercise regulates **neurotransmitter** release. A recent study on humans has revealed that vigorous exercise leads to higher release of glutamate and gamma-Aminobutyric acid (GABA) in executive regions (Maddock et al., 2016). Because of its excitatory function, glutamate is essential to communication among neurons and is at the base of learning processes. Glutamate is the precursor of GABA and its antagonist. GABA inhibits cellular communication and performs regulative functions in neurogenesis, in the growth of neurites, and in the formation of synapses.

Sixth, **mood disturbances** such as depression are known to be detrimental to learning and thus to academic achievement (Esch et al., 2014). Students who practice exercise show less mood disorders and greater propensity to mastering tasks. Matta Mello Portugal et al. (2013) explain this by mechanisms as neurogenesis, angiogenesis, and brain plasticity induced through neurotransmitters and neurotrophic factors, as described in the preceding sections.

In the twenty-first century, these studies among many others provide robust evidence that the Latin saying *mens sana in corpore sano* (a healthy mind in a healthy body) indeed is still a valid principle (Donnelly et al., 2016). Despite this, time spent in physical exercise in the weekly agenda is declining, and young generations are more sedentary than in the past (Biddle et al., 2014). Obesity is seriously menacing the cognitive performance of young generations worldwide (Wang et al., 2016). In fact, obesity has been found to correlate negatively with executive functions, attention, visuo-spatial performance, and motor skills (Liang et al., 2013). Recently, Heyward and colleagues have discovered that obesity induces memory impairment, which is detrimental to academic achievement (Heyward et al., 2016). Progressive governments in Western countries have started to contemplate this and are moving to place physical activity at the center of the school day (Willeboordse et al., 2016). Programs are being tested to reduce weight and enhance cognitive performance (Wright et al., 2016). In practice, novel approaches prove the validity of physical exercise for cognitive performance; in research, empirical studies assess this (Bartholomew and Jowers, 2011; Donnelly and Lambourne, 2011; Norris et al., 2015).

## How the body helps the mind via gestures

In line with the traditional view that body and mind are two separate identities, instruction has not considered employing the body as a tool during learning. Despite this, an increasing number of studies show the tight link between cognitive processes and gestures.

Beaudoin-Ryan and Goldin-Meadow (2014) investigated how children learn **moral reasoning** at school, i.e., how to deal with moral issues such as gun control and the death penalty. At the base of the reasoning are perspective-taking and empathy. In the study, children were told to gesture and not to gesture while explaining their reasoning. The control group did not receive any instruction. Those children who were asked to gesture produced spatial gestures. By doing so, the gesturing children visualized the perspectives that they were taking. The same children outperformed their peers in the results because they were able to assume multiple perspectives while reasoning.

Another domain in which gestures have been demonstrated to ease a cognitive task is **spatial problem solving**. In a series of experiments, Chu and Kita (2011) observed that participants spontaneously gestured when solving mental rotation problems. More interestingly, the researchers found a positive correlation between the number of gestures and the difficulty of the task. Gestures were dropped when the solving strategies had been internalized.

In a study on **working memory**, subjects were asked to remember letters and to accompany them with gestures or meaningless movements while performing another cognitive task. Gestures, i.e., semantic movements, helped subjects to recall the letters better than did meaningless movements (Cook et al., 2012).

Collectively, this research suggests that there is a strong relationship between cognitive effort and gesture use, and that the body spontaneously and consistently helps the mind with a strategy that we have not been overtly taught. Anectodically speaking, German has an expression to describe gesturing in a foreign country when we do not speak the language: speaking with our hands and feet. We all have experienced using a whole range of gestures to fill the gaps of missing words and expressions. We point to objects and use deictic, representative, symbolic gestures, etc. If concepts were abstract symbols and amodal (Fodor, 1983; Pylyshyn, 1984), we would not make use of our body to bridge cognitive gaps and support cognition altogether. We acquire knowledge via our bodily experiences in interaction with the world (Barsalou, 2008). Our representation of knowledge is detectable in the brain and its nature is sensorimotoric and by no means abstract (Gallese and Lakoff, 2005).

In the early 1980s, research demonstrated that performing a gesture to a word/phrase enhances its memorability (Zimmer, 2001). This has been proven also in second **language** in the short and the long term (for a review, see Macedonia, 2014). Among other reasons, the effect of gestures on memory for verbal information is explained in terms of a motor trace left in the concept's representation by the actions performed (Engelkamp and Zimmer, 1985). In an experiment with functional magnetic resonance imaging, participants were presented words that they had previously learned with semantically related gestures and unknown words. Passive recognition of learned words elicited activity in motor cortices and subcortical structures involved in action performance (Macedonia and Mueller, 2016). The authors of the study explain the positive effect of gestures on memory for words with the formation of vast sensorimotor networks (motor trace) connecting declarative and procedural memory.

In **mathematics**, hand gestures have also been shown to have a beneficial effect on learning. Cook et al. (2016) made an avatar explain mathematical equivalence to school children with and without (beat and representational) gestures. In the gesture condition, children had better performance in problem solving than their peers in the non-gesture condition. Moreover, the former were faster than the latter. These results are in line with a study by Alibali and Nathan (2011) that explains the effect of representational gestures as “mental simulations of actions.” These actions enact concepts and make them more understandable (Crooks and Alibali, 2014). We agree with Radford (2009) that “*the very texture of thinking … cannot be reduced to impalpable ideas; it is instead made up of speech, gestures, and our actual actions with cultural artifacts (signs, objects, etc.)*”.

Language and mathematics provide examples that the body can be easily used as a learning tool. Specifically, as gestures are the natural, and spontaneous tool that connects body and mind, gestures should be integrated in teaching methods and classroom activities: since Roth (2001) made this suggestion, little has happened in this regard. Education is still reluctant, despite publications for practice with concrete descriptions of how to use gestures as a tool to enhance second language vocabulary acquisition (Macedonia, 2013, 2014; Macedonia and Klimesch, 2014).

In education the body must be taken into consideration and actively involved in the learning process.

## Author contributions

MM as a first author has written the paper. CR as a second and last author has contributed with discussions, literature, and with editing of the paper.

### Conflict of interest statement

The authors declare that the research was conducted in the absence of any commercial or financial relationships that could be construed as a potential conflict of interest.
